# Comparison of *in vitro* and computational experiments on the relation of inter-beat interval and duration of repolarization in a specific type of human induced pluripotent stem cell-derived cardiomyocytes

**DOI:** 10.1371/journal.pone.0221763

**Published:** 2019-09-09

**Authors:** Philipp Kügler, Georg Rast, Brian D. Guth

**Affiliations:** 1 Institute of Applied Mathematics and Statistics, Computational Science Lab, University of Hohenheim, Stuttgart, Germany; 2 Department of Drug Discovery Sciences, Boehringer Ingelheim Pharma GmbH & Co. KG, Biberach an der Riss, Germany; 3 Department of Pharmaceutical Sciences, North-West University, Potchefstroom Campus, Potchefstroom, South Africa; University of Oxford, UNITED KINGDOM

## Abstract

We compared a published computational model of the action potential of a specific type of human induced pluripotent stem cell -derived cardiomyocytes (hiPSC-CM) with experimental field potential data with regard to their inter-beat interval and the duration of repolarization. In particular, concomitant changes in inter-beat interval and duration of repolarization were calculated after reduction and/or augmentation of specific ion channel conductances as a surrogate for pharmacological manipulation. The observed mismatches between calculations and experimental data indicate that there is information missing about the cellular test system. Based on our results we hypothesize that, among other currents, the actual I_f_ (“funny current”) may deviate from the prediction. We show that replacement of the I_f_ formulation by alternative equations causes the model predictions to change qualitatively, however, none of the available formulations is actually achieving a satisfactory match with experimental data. We suggest a strategy to clarify whether the mismatch can be completely resolved at all using single cell models and, if yes, how this goal could be reached.

## Introduction

Computational models of biological systems are based both on theoretical concepts and experimental data, but also to some extent on assumptions. Simulations may be used to obtain experimentally inaccessible results or to predict experimental conditions, like ion concentrations, to achieve specific model system properties (e.g. sensitivity to proarrhythmia). However, models must first be validated based on real-life experiments. Mismatches between real-life and computational experiments may not only occur simply due to uncertainties in the model parameters or even the model’s structure, but they may also indicate a lack of understanding of the underlying biology.

In various types of hiPSC-CM the typical dependency of repolarization on inter-beat interval known from *in vivo* studies or *ex vivo* experiments with cardiac tissue is replaced by a complex mutual interdependency [1]. For example, pharmacological interventions that only prolong repolarization *in vivo* also prolong the inter-beat interval in hiPSC-CM [2–4]. This ambiguity is often ignored or even inadequately “corrected”, although its impact on the interpretation of repolarization data in spontaneously beating cells may be considerable. Indeed, in many cases, mechanisms that affect inter-beat interval and mechanisms that affect repolarization may not be distinguishable at all [1]. However, inadequate “correction” may unduly tip the scales into either direction, leading to faulty data interpretation, mostly in the context of pro-arrhythmic risk assessment for which repolarization metrics are commonly used as surrogate parameters.

A published computational model of hiPSC-CM [5–7] has been constructed to faithfully predict action potentials (APs) under undisturbed conditions and some specific interventions [8] and to date represents the only published computational model of iCells (Cellular Dynamics International, Madison, WI, USA, a Fujifilm Company). However, it has not been specifically optimized for the prediction of the interrelation of repolarization and inter-beat interval, although it is built on experimental data mostly coming from the cell type we used to empirically characterize this effect. We therefore checked whether this interrelation is already integrated in the model. For this purpose we reconstructed experiments with pharmacological interventions that are believed to primarily affect inter-beat interval (modulation of I_f_) and repolarization (inhibition of IK_r_ and ICa_L_) based on the empirical phenotype of reference compounds addressing these targets.

Misprediction of such experimental data demonstrates that the model is missing some features that only become apparent when it is challenged with surrogates for the pharmacological interventions that reveal the interrelation of repolarization and inter-beat interval. We tested the influence of different mathematical formulations of I_f_ [9–11] on this inter-relation, but only obtained minor improvements. We therefore discuss strategies to better understand the inter-relation of spontaneous rate and repolarization in iCells and how this could help to develop a computational model which reflects this inter-relation.

## Materials and methods

### Computational models

All simulations were run with initial conditions as published previously [5, 6] until limit cycle behavior was reached (5000 s); after perturbation, simulations were run for further 5000 s and the last 1000 seconds were analyzed. In order to assess acute effects, in some cases the perturbation was placed at the minimum potential between two action potentials and the first and second action potential after perturbation were analyzed. Inter-beat intervals (“rr”, written in lower case to underscore the distinction from the RR interval taken from an ECG) were calculated using the Matlab routine “findpeaks” for the detection of local maxima of the voltage trace. Given a model for a spontaneously beating AP, we chose the moment of the maximum upstroke velocity as the initial time of the AP for the calculation of APD90. Finally, the rr and APD90 sequences were averaged in order to reduce the impact of numerical round off errors. All calculations were done in Matlab R2017b on a Linux 3.12.67-64-desktop with 8 Intel Xeon E5-2687W 3.10 GHz 20MB CPUs. In order to speed up the numerical integration of the stiff ordinary differential equation systems, we compiled a modified Matlab code of the published models [5–7] ([5] with modifications up to 2017 [6] referred to as Paci2017 and [7] as Paci2018) and used the SUNDIALS solver CVODE. The original Matlab codes of the models were kindly provided by the authors of [5–7].

### Experimental data

The experimental data referred to in this study were taken from [12] and [1] as indicated in the respective figure legends. Briefly, field potential recordings were taken from confluent monolayers of iCells (Cellular Dynamics International, Madison, WI, USA, a Fujifilm Company) using the USB-MEA 256 multi-electrode array recording system (Multichannel Systems, Reutlingen, Germany, a division of Harvard Bioscience) as described in [12]. The inter-beat interval (“rr”) was calculated from thresholding on the fast component of the field potential and the duration of repolarization was measured as the time from threshold to the maximum of the slow repolarization component of the field potential (“qtmax”). As for “rr” the term “qtmax” is written in lower case to underscore the distinction from the QT interval taken from an ECG. The relation of rr versus qtmax was modelled as a second order polynomial using Prism software (GraphPad, La Jolla, CA, USA) [1].

### Simulation protocols

**Fig 1:**
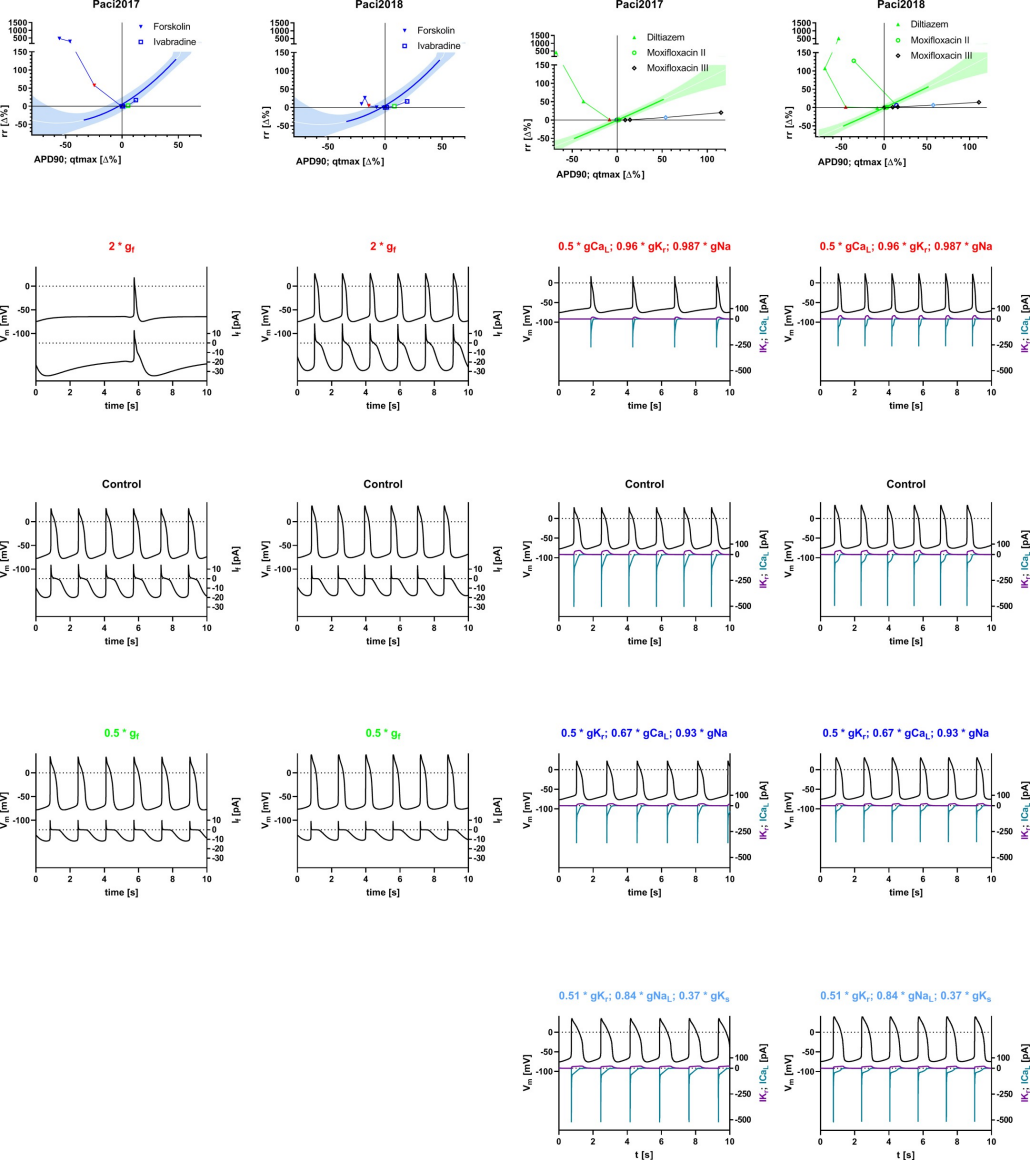
Inter-relation of inter-beat interval (rr) and repolarization (APD90 for simulations, qtmax for experimental data) after modulation of I_f_, IK_r_, and ICa_L_ with Ivabradine, Forskolin, Diltiazem, and Moxifloxacin, including reported off-targets. First row: Blue: Data relating to mechanisms primarily affecting rr (reduction and augmentation of I_f_ with Ivabradine and Forskolin). Green: Data relating to mechanisms primarily affecting repolarization (reduction of ICa_L_ or IK_r_ with Diltiazem and Moxifloxacin). Shaded areas: 90% prediction intervals for polynomial fit (solid lines) of experimental data as published in [1]; solid green and blue lines within shaded areas indicate dynamic range of experimental data. The graphical representation of experimental data is modified from [1] and for illustrative purposes only. Symbols: simulated data; filled inverted triangles: augmentation of I_f_; open squares: reduction of I_f_; filled upright triangles: reduction of ICa_L_ (plus IK_r_ and INa as off-targets of Diltiazem, see Table 1); open circles: reduction of IK_r_ (plus ICa_L_ and INa as off-targets of Moxifloxacin, see Table 2); open diamonds: reduction of IK_r_ (plus IK_s_ and INa (late) as alternative off-targets of Moxifloxacin, see Table 3). Second to fifth row: Simulated action potentials (top, left y-axis) and currents (bottom, right y-axis) with doubling of g_f_ with Forskolin or half inhibition of gCa_L_ with Diltiazem (plus off-targets, second row), no modification of conductances (control, third row), half inhibition of g_f_ with Ivabradine or gK_r_ with Moxifloxacin (plus off-targets according to Table 2, fourth row) and half inhibition of gK_r_ with Moxifloxacin (plus off-targets according to Table 3, fifth row). Scale factors given in the titles of the panels correspond to the symbols in the first row graphs with same color. When two different currents are plotted on a common y-axis, the trace color corresponds to the color of the axis label. First and third column: Model from [5] with modifications until 2017 [6] (Paci2017). Second and fourth column: Model from [7] (Paci2018).

We checked whether the computational model was able to reproduce the dependency of inter-beat interval (rr) on duration of repolarization (qtmax) specific for iCells, as described in [1] (Fig 1). To simulate pharmacological modulation of ICa_L_ with Diltiazem in the concentration range used experimentally, the maximal permeability (parameter gCa_L_ [5], supplement) was modified as outlined in Table 1. Off-target ion channel inhibition was taken into account according to selectivity data given in [13], supplement (see also Table 1). Likewise, the maximal conductance of IK_r_ (parameter gK_r_ [5], supplement) was modified as outlined in Table 2 and Table 3 to simulate IK_r_ inhibition with Moxifloxacin in the experimental concentration range, also including reported off-target ion channel inhibition [13], supplement. To evaluate the impact of inter-beat interval changes, maximal conductance of I_f_ (parameter g_f_ [5], supplement) was set to 0%, 50%, 90%, 99%, 101%, 110%, 150%, 200%, 300%, and 400%, of its original value. These changes were arbitrary as the experimental results with Ivabradine had been obtained with a single low concentration (0.3 μM) to ensure selectivity. Incremental effects resulted from incremental incubation periods. Forskolin is an indirect activator of I_f_ and no concentration effect relation for iCells is available.

**Table 1:** Scale factors used to simulate pharmacological manipulation with Diltiazem.

IC_50_ = 0.76 μM Hill slope = 1.14	Diltiazem	IC_50_ = 13.2 μM Hill slope = 1.16	IC_50_ = 22.4 μM Hill slope = 1.29
Scale factor gCa_L_	Concentration [μM]	Scale factor gK_r_	Scale factor gNa
0.05	10.1	0.58	0.74
0.1	5.22	0.75	0.87
0.5	0.76	0.96	0.987
0.9	0.111	0.996	0.999

Scale factors for the primary target (gCa_L_, first column) were chosen to cover a reasonable range and theoretical concentrations (second column) were calculated according to IC_50_ and Hill slope (first and second line) from [13], supplementary material (“Diltiazem I”). Resulting scale factors for the off-targets (third and fourth column) were calculated according to IC_50_ and Hill slope from the same source.

**Table 2:** Scale factors used to simulate pharmacological manipulation with Moxifloxacin.

IC_50_ = 86.2 μM Hill slope = 0.94	Moxifloxacin	IC_50_ = 173 μM Hill slope = 1	IC_50_ = 1112 μM Hill slope = 1
Scale factor gK_r_	Concentration [μM]	Scale factor gCa_L_	Scale factor gNa
0.25	277.4	0.38	0.80
0.5	86.2	0.67	0.93
0.9	8.32	0.95	0.993
0.95	3.76	0.979	0.997

Scale factors for the primary target (gK_r_, first column) were chosen to cover a reasonable range and theoretical concentrations (second column) were calculated according to data from [13], supplementary material (“Moxifloxacin II”), repeated in first line of table. Resulting scale factors for the off-targets (third and fourth column) were calculated according to data from the same source.

**Table 3:** Alternative scale factors used to simulate pharmacological manipulation with Moxifloxacin.

IC_50_ = 93.041 μM Hill slope = 0.6	Moxifloxacin	IC_50_ = 382.337 μM Hill slope = 1.1	IC_50_ = 50.321 μM Hill slope = 1
Scale factor gK_r_	Concentration [μM]	Scale factor gNa_L_	Scale factor gK_s_
0.34	277.4	0.59	0.15
0.51	86.2	0.84	0.37
0.81	8.32	0.985	0.86
0.87	3.76	0.994	0.930

Scale factors for the primary target (gK_r_, first column) were chosen to cover a reasonable range and theoretical concentrations (second column) were calculated according to data from [13], supplementary material (“Moxifloxacin III”), repeated in first line of table. Resulting scale factors for the off-targets (third and fourth column) were calculated according to data from the same source.

## Results

### rr versus APD90 relation

The primary effects on rr obtained by experimentally inhibiting or augmenting g_f_ (with Ivabradine and Forskolin) could neither be reproduced with Paci2017 nor with the updated version Paci2018 [5–7] under limit cycle conditions (Fig 1, first and second column). The Matlab code for the generation of the simulation data can be found in the supporting information (S1 Code) along with the parameters for the reference curve fits and the actual tabulated result values (S1 Table). In the simulations, both inhibition and augmentation caused a prolongation of rr in the models to various extents, whereas, experimentally, augmentation of g_f_ with Forskolin caused a reduction of rr in line with the commonly assumed physiological role of I_f_ [14, 15]. For comparison, acute effects of augmentation or reduction of g_f_ are shown in S1 Fig (first row) and tabulated in S3 Table. In some instances acute effects point in the opposite direction than under limit cycle conditions (e.g. S1 Fig, middle and right panel). In both model versions, inhibition of g_f_ caused a prolongation of APD90, an effect which is in line with experimental data and is commonly explained to be secondary to the prolongation of rr. However, in the simulations of I_f_ augmentation, rr was unexpectedly prolonged. Surprisingly, APD90 was simultaneously reduced, an effect which cannot be rationalized as being secondary to rr prolongation.

In both model versions, the reduction of gK_r_ (plus reduction of gCa_L_ and gNa as off-targets of Moxifloxacin, see Table 2) resulted only in a minimal prolongation of APD90 and in case of Paci2018 with maximal gK_r_ inhibition in even a reduction of APD90. This does not match with the commonly assumed physiological role of IK_r_ and experimental results [16, 1]. Only a minimal prolongation of inter-beat-interval was associated with this effect, except for Paci2018 with maximal inhibition IK_r_ and the off-targets associated with Moxifloxacin according to Table 2. This is compatible with *in vivo* data [17–20], but in hiPSC-CM, a prolongation of rr is usually observed [1–4]. In contrast, when an alternative off-target profile of Moxifloxacin is used for simulations (Table 3) a prolongation of APD90 is predicted which exceeds the magnitude observed in experimental data at comparable concentrations. However, the experimentally observed prolongation of rr is still consistently under-predicted. The reduction of gCa_L_ (plus reduction of gK_r_ and gNa as off-targets of Diltiazem, see Table 1) resulted in a reduction of APD90 which is in line with experimental results both in hiPSC-CM and primary cardiomyocytes [21, 22, 1]. rr is prolonged under these conditions in both model versions, however experimentally, both prolongation and reduction may be observed depending on the specific vendor of the hiPSC-CM [1]. In iCells, the cell type from which most experimental data for the Paci2017 and Paci2018 models were derived, a reduction is consistently observed [1].

### Formulation of I_f_

**Fig 2:**
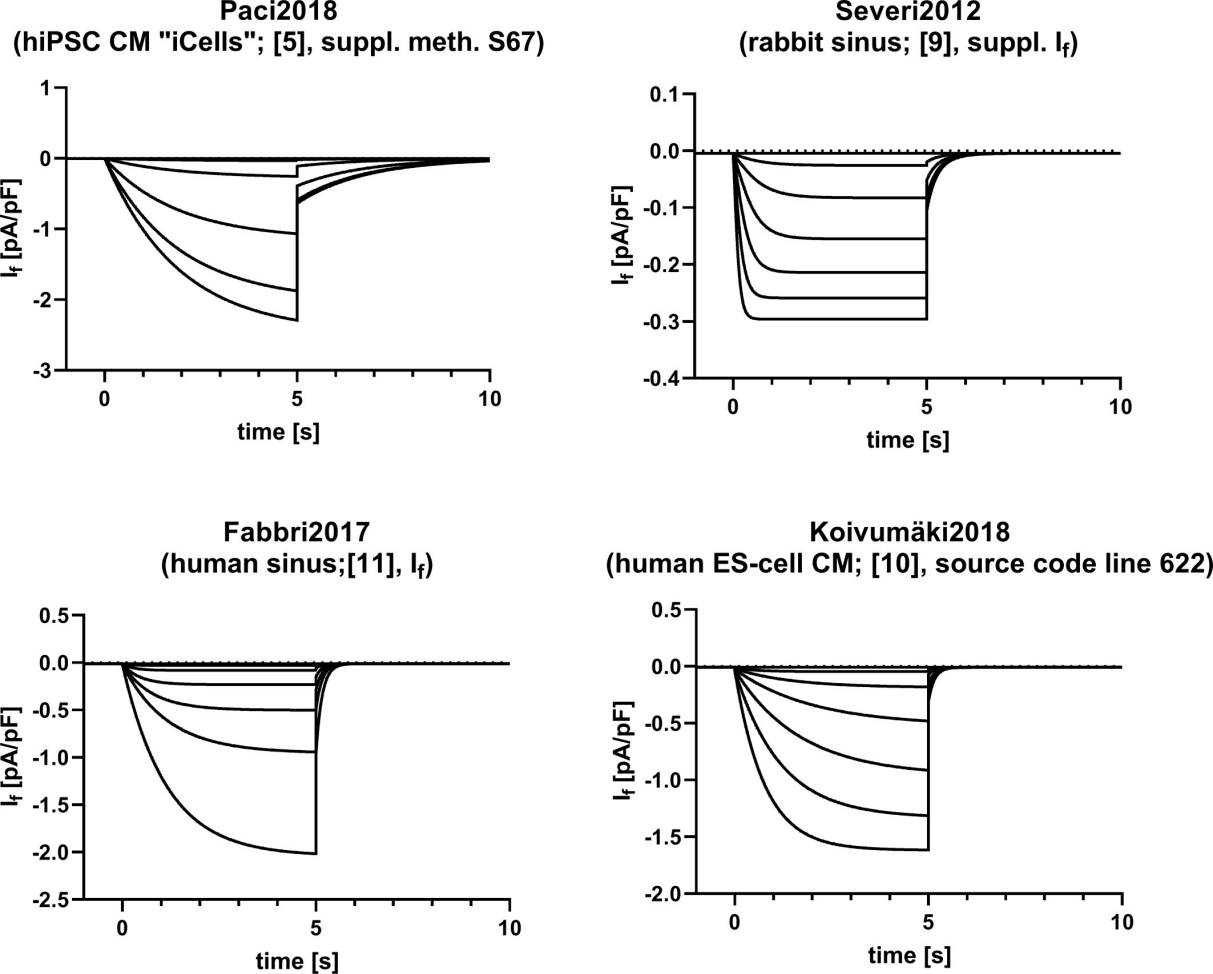
I_f_ in a simulated voltage clamp protocol using various current formulations. I_f_ starting at -40 mV with hyperpolarizing steps to -50 - -100 mV (in 10 mV increments) for 5 s, followed by a step back to -40 mV for another 5 s. Calculations have been performed using the “blank” cellular model of [5], i.e. membrane capacitance, g_f_, and both internal and external ionic concentrations from [5] were used for all I_f_ variants. Top: I_f_ from [5] (Paci2018, left) and [9] (Severi2012, right); bottom: I_f_ from [11] (Fabbri2017, left) and [10] (Koivumäki2018, right).

As experimental data clearly demonstrate the influence of I_f_ on spontaneous rate in iCells, we tested whether alternative published mathematical formulations of I_f_ [9–11] would improve physiological accuracy of the hiPSC-CM model. The formulations differ substantially with regard to gating behavior, both in terms of voltage dependence and kinetics (Fig 2). In all formulations g_f_ was set to the value given in [5] to make sure that the virtual channel density remains the same for all comparisons.

**Fig 3:**
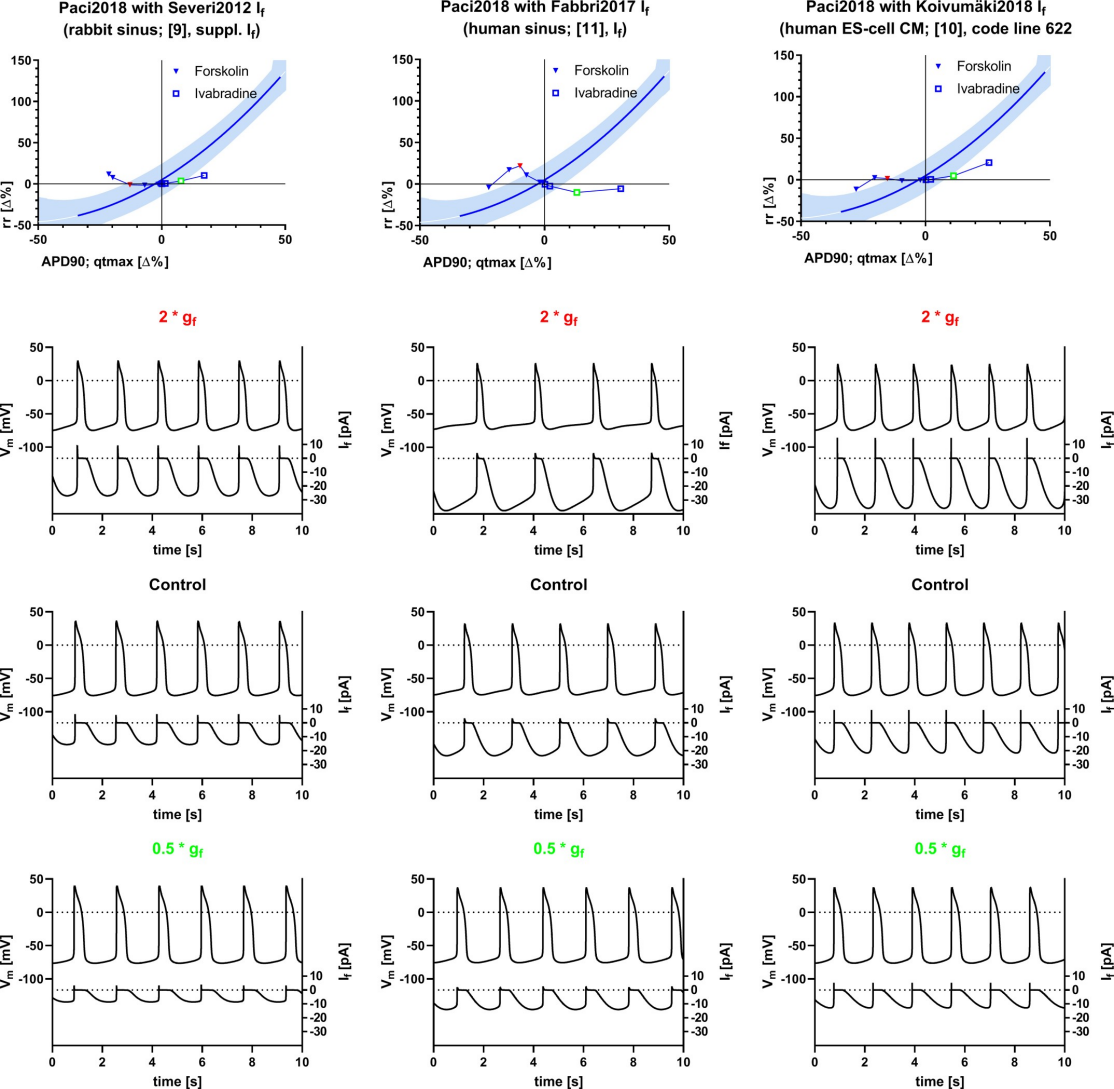
Inter-relation of rr and repolarization (APD90 for simulations, qtmax for experimental data) after modulation of g_f_ in the Paci2018 [7] model with modified I_f_. I_f_, in the model from [7] was substituted with the equation for I_f_ from [9] (Severi2012, left column), [11] (Fabbri2017, middle column), and [10] (Koivumäki2018, right column). For an explanation of colors, symbols, and simulated traces see Fig 1. The graphical representation of experimental data is modified from [1] and for illustrative purposes only.

The same computational experiments as for the original models were performed under limit cycle conditions with versions of Paci2018 with substitutions of the I_f_ formulation with the ones shown in Fig 2 (Fig 3). The Matlab code for the generation of the simulation data can be found in the supporting material (S1 Code) along with the parameters for the reference curve fits and the actual tabulated result values (S2 Table). For comparison, acute effects of g_f_ reduction or augmentation in the modified models are shown in S1 Fig (second row) and tabulated in S3 Table. A complementary figure illustrating reduction of gCa_L_ and gK_r_ (plus the respective off-targets of Diltiazem and Moxifloxacin) in the modified model versions can be found in the supporting material (S1 Fig), along with the actual tabulated result values (S3 Table).

In all three modifications of the Paci2018 model, reduction or augmentation of g_f_ (as experimentally performed with Ivabradine and Forskolin) causes effects that qualitatively match the experimentally observed effects on qtmax, however, the dynamic range is substantially smaller than observed experimentally, particularly at full g_f_ inhibition and the influence on rr is not correctly predicted almost throughout (Fig 3). As effects on repolarization are expected to be secondary to effects on rate in this context, we have to state that all model versions analyzed with respect to limit cycle behavior fail to predict the experimental results, however, in the versions with a substitution of I_f_ with formulations fitted to experimental data from rabbit sinus cells [9] and human ESC CMs [10] the acute effects on rr are predicted correctly (S1 Fig, second row, left and right panel and S3 Table).

Reduction of gCa_L_ (plus gKr and gNa as off-targets of Diltiazem, see Table 1), in the modified model versions, leads to a reduction of APD90 as expected and predicted by the previous model versions. The secondary effect on inter-beat-interval in this case assumes an intermediate position between the effects seen for different vendors. At low reduction values up to ca. 50% gCa_L_ (plus off-targets) a decrease of rr as in iCells is seen, though much less pronounced than expected. However, at almost full inhibition (plus off-targets) a prolongation of rr is observed (S2 Fig, S4 Table).

Similar to the original model versions, only a minimal prolongation of rr was associated with a prolongation of APD90 as a response to reduction of gK_r_ (plus gCa_L_ and gNa as off-targets of Moxifloxacin, see Table 2) in the model versions with alternative I_f_ formulations. However, at the strongest reduction of gK_r_ and the off-targets associated with Moxifloxacin, the effect was reverted to a reduction of APD90 and a prolongation of rr (S2 Fig, S4 Table). As in the original model versions, an alternative off-target profile of Moxifloxacin (Table 3) causes a prolongation of APD90 exceeding the magnitude observed in experimental data at comparable concentrations and under-predicts the experimentally observed prolongation of rr (S2 Fig, S4 Table).

## Discussion

This study suggests that the *in silico* hiPSC-CM model [5–7], which recapitulates the action potential observed in iCells, does not predict concomitant changes of qtmax and rr that have been reported for this cell type in vitro [1] for specific pharmacological interventions. The expected reduction or prolongation of inter-beat interval after augmentation or reduction of g_f_ (as experimentally performed with Ivabradine and Forskolin) is not predicted under limit cycle conditions and no substantial improvements could be obtained with different formulations for I_f_. However, in two modifications of the Paci2018 model [7] (substitution of I_f_ with formulations fitted to experimental data from rabbit sinus cells [9] and human ESC CMs [10]) acute changes in rr after augmentation or reduction of g_f_ are predicted correctly, indicating slow adaptive processes over-compensating the initial response under limit cycle conditions. Furthermore, in iCells mechanisms beyond I_f_ may contribute to automaticity, however, these are not characterized, yet. In the family of computational models published specifically for iCells [5–8], a contribution of the large sodium window current is suggested [10].

The experimentally observed prolongation of rr associated with a prolongation of qtmax after reduction of gK_r_ (plus off-targets, as experimentally performed with Moxifloxacin, see Tables 2 and 3) is consistently under-predicted. Furthermore, for strong inhibition of gK_r_ the expected prolongation of APD90 is predicted to be reversed for a specific off-target profile of Moxifloxacin. This is likely due to a concomitant reduction of ICa_L_ because of the lack of selectivity reported for Moxifloxacin in [13] (“Moxifloxacin II”, see also Table 2) at this concentration. The experimental results reported in [1] for similar concentrations of Moxifloxacin do not support this prediction. It needs to be stated that the IC_50_ values and Hill slopes listed in [13] for “Moxifloxacin II” (Table 2) and used for this simulation deviate substantially from other sources, like [23], and an alternative profile listed in [13] (“Moxifloxacin III”, Table 3) lacking ICa_L_ inhibition. This alternative profile consistently causes the expected prolongation of APD90, however, still under-predicts effects on rr. This example underscores how simulations are limited by the accuracy and comprehensiveness of supporting experimental data.

The reduction of qtmax or APD90 after reduction of ICa_L_ (plus off-targets, as experimentally performed with Diltiazem, see Table 1) is predicted correctly throughout, however, the concomitant reduction of rr, as consistently observed in iCells [1], is not captured by any model version. Rather, rr tends to be prolonged to varying degrees. This can experimentally be observed in other cell types, e.g. Pluricytes [1].

### Limitations

Although the experimental data used for comparison have been produced with the very same cell line as most of the data used for the construction of the *in silico* model, some limitations apply: qtmax used as a read-out for the duration of repolarization in experimental data differs from APD90 in the simulation. However, we consider the relationship between APD90 and qtmax as rigid enough that the parameters may be used interchangeably in our context [24]. Experimental field potential data were always obtained from confluent monolayer cultures of cells whereas the simulations apply to a single cell action potential. This has the following implication: in case effects are brought about by cellular mechanisms, meaningful conclusions may be drawn from a single cell model. However, in case effects are due to heterogeneity and cellular coupling, a different and more complex modelling approach should be selected. In the latter case, the effects seen on qtmax may come from the cells closest to the field electrode whereas the effects on rr may originate from a potentially tiny and remote subpopulation of different cells pacing the whole culture. In this case it is obvious that both parameters, qtmax and rr, cannot not be predicted correctly by a single cell model.

Whether iCell monolayers are actually that heterogeneous, needs to be clarified with careful single cell characterizations encompassing a sufficient sample size. Existing studies already suggest a considerable heterogeneity [25], however, it is not clear yet whether the heterogeneity is sufficient to explain the discrepancy between multi-cellular systems and a single cell simulation.

Experimental studies on single cells with regard to their inter-beat interval and repolarization time are technically demanding and require sparsely seeded cells, unless the approach of pharmacological de-coupling in a confluent monolayer as in [25] is chosen. In this publication it is suggested that sparsely seeded cells have different electrophysiological properties than when seeded as a confluent monolayer, however, a complete de-coupling of such monolayers is difficult to achieve due to the lack of sufficiently potent and selective gap junction blockers. Therefore, both possible approaches to study single cell electrophysiology, sparsely seeded cells and pharmacologically decoupled cells in a confluent monolayer, suffer from potential limitations.

Experimental pharmacology always has to deal with potential selectivity issues of the tool compounds used. For the experiments performed in [12] and [1] compounds were used at concentrations assumed to be sufficiently selective. Forskolin, however, is a non-specific activator of adenylyl cyclase and therefore may have multiple cellular effects beyond activation of I_f_, e.g. activation of IK_s_ and hence a reduction of qtmax. Since in our study we wanted to analyze the impact of rr on qtmax and vice versa, the mechanism by which rr is affected is less important than making sure that the mechanism of choice has no relevant direct impact on qtmax. In guinea pig papillary muscle it has been shown that a water-soluble derivative of Forskolin (NKH-477) has little impact on repolarization at concentrations relevant for this study [26], however, in rabbit papillary muscle Forskolin was reported to cause a mild reduction of APD90 of about 11% in the relevant concentration range [27]. In iCells, the stimulating effect of Forskolin on I_f_ was counter-balanced by co-application of Ivabradine and no relevant residual reduction of qtmax was seen that could be attributed to a direct effect of Forskolin on repolarization (S3 Fig).

The simulation of ion channel inhibition by scaling the conductance does not take into account dynamic effects (e.g. state- or voltage-dependent affinity shifts with associated kinetics), however, this method has proven to be an effective approach [8]. Moreover, the simulation of dynamic effects would require the use of Markov-type models for each ion channel which are not available for all the channels included in the Paci et al. hiPSC-CM model of iCells.

### Inter-relation of repolarization and inter-beat interval

The exact mechanism of the dependence of repolarization on inter-beat interval *in vivo* and *ex vivo* is not known and likely to be complex, as lag times and hysteresis are broadly variable [28]. A dependency of rr on qtmax is so far only described for hiPSC-CM, particularly iCells [1], and in the sino-atrial node e.g. [29]. It has been suggested [1] that rr in iCells may be limited by the relative refractory period of the cells which is determined by qtmax. This implies that the depolarizing drive is strong enough in these cells to elicit an action potential as soon as recovery from refractoriness permits. It should be possible to implement such a hypothetical mechanism in a single cell model, however, our results obtained with the computational models tested, suggest that this potential mechanism is so far not represented *in silico*, as prolongation of APD90 by IK_r_ reduction (plus off-targets, as experimentally performed with Moxifloxacin, see Tables 2 and 3) has much less impact on inter-beat interval than it has experimentally (Fig 1). Moreover, replacing the formulation of I_f_ with qualitatively different equations does not change this shortcoming (Fig 3).

As reduction of g_f_ under-predicts the experimental effects on rr of Ivabradine in all model variants no conclusion can be drawn whether the expected adaptive prolongation of APD90 can be expected in the models. For an augmentation of g_f_, the expected reduction of rr is even reversed under limit cycle conditions. Therefore, acute effects were analyzed and a correct prediction of rr reduction was achieved. However, in this case, it is impossible to judge the concomitant APD90 changes, as these are expected to be adaptive in response to the rr change and therefore only consistently accessible when a limit cycle has been reached.

Generally, this study is limited to a family of computational models that have been tailored to experimental data from iCells [5–8], however, further computational models exist which are based on different hiPSC-CM, e.g. [10], for which this mechanism has not been analyzed.

Despite of technical limitations and an insufficient database on the potential heterogeneity of iCells we propose the following strategy to clarify whether the specific inter-relationship of rate and repolarization in iCells is due to heterogeneity in a multicellular system or a cellular phenomenon:

1. As laid out above, both sparsely seeded cells and de-coupled monolayers have their limitations but they may serve as references for the two extremes of a spectrum. In both settings it needs to be established which spectrum of cellular phenotypes exists that may encompass potentially few cells which drive automaticity with little impact on field potential duration and other cells that rarely beat spontaneously but determine field potential duration. Once the phenotypic spectrum is characterized, representative model variants would have to be established and integrated into multicellular models with hypothetical spatial distributions. A study with coupled atrial-like and ventricular-like variants of the Paci2018 model has been performed [30], however, the inter-relationship of rate and duration of repolarization has not been analyzed in this setting.
2. Alternatively, the work presented in this study needs to be continued to establish whether a refined model is capable of quantitatively predicting the inter-relationship of rate and repolarization in a single cell. Manipulating sodium channel recovery from inactivation both experimentally and in the computational model should indicate whether the hypothesis of refractoriness controlling spontaneous rate can be further maintained.

## Supporting information

S1 Code Matlab code for the computational models used for Fig 1 and Fig 3. (ZIP)

S1 Table Tabulated results visualized in Fig 1. (XLSX)

S2 Table Tabulated results visualized in Fig 3. (XLSX)

S3 Table Tabulated results visualized in S1 Fig. (XLSX)

S4 Table Tabulated results visualized in S2 Fig. (XLSX)

S1 Fig Comparison of acute effects of g_f_ disturbance on rr and APD90 with limit cycle behavior as shown in Figs 1 and 3. Acute effects were analyzed as the rr interval between the first and the second beat after a change in g_f_ introduced at the minimum potential within an inter-beat interval. The corresponding APD90 was taken from the first action potential after the disturbance. First row: Comparison of acute and limit cycle effects for the unchanged models Paci2017 and Paci2018 (left and middle) and an example graph for the course of rr intervals during 5000 subsequent cycles in the Paci2018 model with a triplication of g_f_ at the inter-beat interval marked by a vertical line. The red symbols in the middle rr vs APD90 graph correspond with the right panel. Second row: Comparison of acute and limit cycle effects for Paci2018 with modified I_f_. The model from [7] (Paci2018) was substituted with the equations for I_f_ from [9] (Severi2012, left), [11] (Fabbri2017, middle), and [11] (Koivumäki2018, right). Shaded areas: 90% prediction intervals for polynomial fit (solid lines) of experimental data as published in [1]; solid blue lines within shaded areas indicate dynamic range of experimental data. The graphical representation of experimental data is modified from [1] and for illustrative purposes only. Symbols: simulated data; filled inverted triangles: augmentation of I_f_; open squares: reduction of I_f_; blue: limit cycle; black: acute.
(TIF)

S2 Fig Inter-relation of rr and repolarization (APD90 for simulations, qtmax for experimental data) after modulation of ICa_L_ and IK_r_ with Diltiazem and Moxifloxacin, including reported off-targets in the Paci2018 model [7] with modified I_f_. I_f_ in the model from [7] was substituted with the equations for I_f_ from [9] (Severi2012, left column), [11] (Fabbri2017, middle column), and [11] (Koivumäki2018, right column). First row: Shaded areas: 90% prediction intervals for polynomial fit (solid lines) of experimental data as published in [1]; solid green lines within shaded areas indicate dynamic range of experimental data. The graphical representation of experimental data is modified from [1] and for illustrative purposes only. Symbols: simulated data; filled upright triangles: reduction of ICa_L_ with Diltiazem (plus IK_r_ and INa as off-targets, see Table 1); open circles: reduction of IK_r_ with Moxifloxacin (plus ICa_L_ and INa as off-targets, see Table 2); open diamonds: reduction of IK_r_ with Moxifloxacin (plus IK_s_ and INa (late) as alternative off-targets, see Table 3). Second to fifth row: Simulated action potentials (top, left y-axis) and currents (bottom, right y-axis) with half inhibition of gCa_L_ with Diltiazem (plus off-targets, second row), no modification of conductances (control, third row),half inhibition of gK_r_ with Moxifloxacin (plus off-targets according to Table 2, fourth row), and half inhibition of gK_r_ with Moxifloxacin (plus off-targets according to Table 3, fifth row). Scale factors given in the titles of the panels correspond to the symbols in the first row plots with same color. When two different currents are plotted on a common y-axis, the trace color corresponds to the color of the axis label. The Matlab code for the generation of the simulation data can be found in the supporting material (S1 Code) along with the parameters for the reference curve fits and the actual tabulated result values are provided in S4 Table.
(TIF)

S3 Fig Lack of a relevant direct effect on qtmax of Forskolin. Averaged field potentials recorded from iCells at baseline conditions (Control, black), with 0.3 μM Forskolin (blue) and with 0.3 μM Forskolin + 0.3 μM Ivabradine (red). As expected, Forskolin shortens rr and concomitantly qtmax; after addition of Ivabradine, the reduction of rr is almost completely reversed as is the reduction of qtmax, indicating no relevant residual direct effect on qtmax of Forskolin.
(TIF)
