# Supplementary figures and images for: Comparison of in vitro and computational experiments on the relation of inter-beat interval and duration of repolarization in a specific type of human induced pluripotent stem cell-derived cardiomyocytes
Source: PLoS One. 2019 Sep 9;14(9):e0221763. doi: 10.1371/journal.pone.0221763 (PMC6733510; doi:10.1371/journal.pone.0221763)

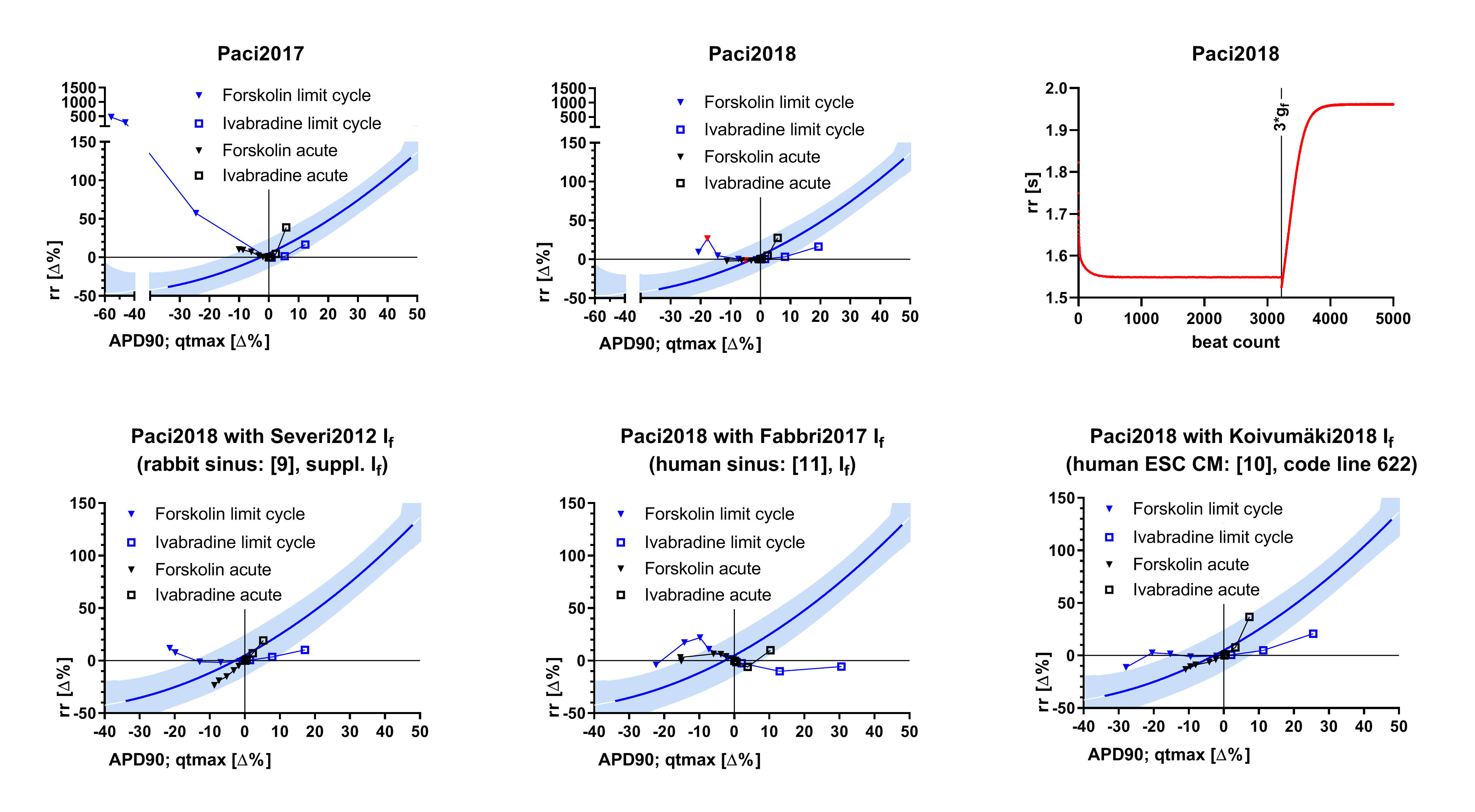

Supplement: S1 Fig — Acute effects were analyzed as the rr interval between the first and the second beat after a change in gf introduced at the minimum potential within an inter-beat interval. The corresponding APD90 was taken from the first action potential after the disturbance. First row: Comparison of acute and limit cycle effects for the unchanged models Paci2017 and Paci2018 (left and middle) and an example graph for the course of rr intervals during 5000 subsequent cycles in the Paci2018 model with a triplication of gf at the inter-beat interval marked by a vertical line. The red symbols in the middle rr vs APD90 graph correspond with the right panel. Second row: Comparison of acute and limit cycle effects for Paci2018 with modified If. The model from [7] (Paci2018) was substituted with the equations for If from [9] (Severi2012, left), [11] (Fabbri2017, middle), and [11] (Koivumäki2018, right). Shaded areas: 90% prediction intervals for polynomial fit (solid lines) of experimental data as published in [1]; solid blue lines within shaded areas indicate dynamic range of experimental data. The graphical representation of experimental data is modified from [1] and for illustrative purposes only. Symbols: simulated data; filled inverted triangles: augmentation of If; open squares: reduction of If; blue: limit cycle; black: acute. (TIF) [file pone.0221763.s006.tif]

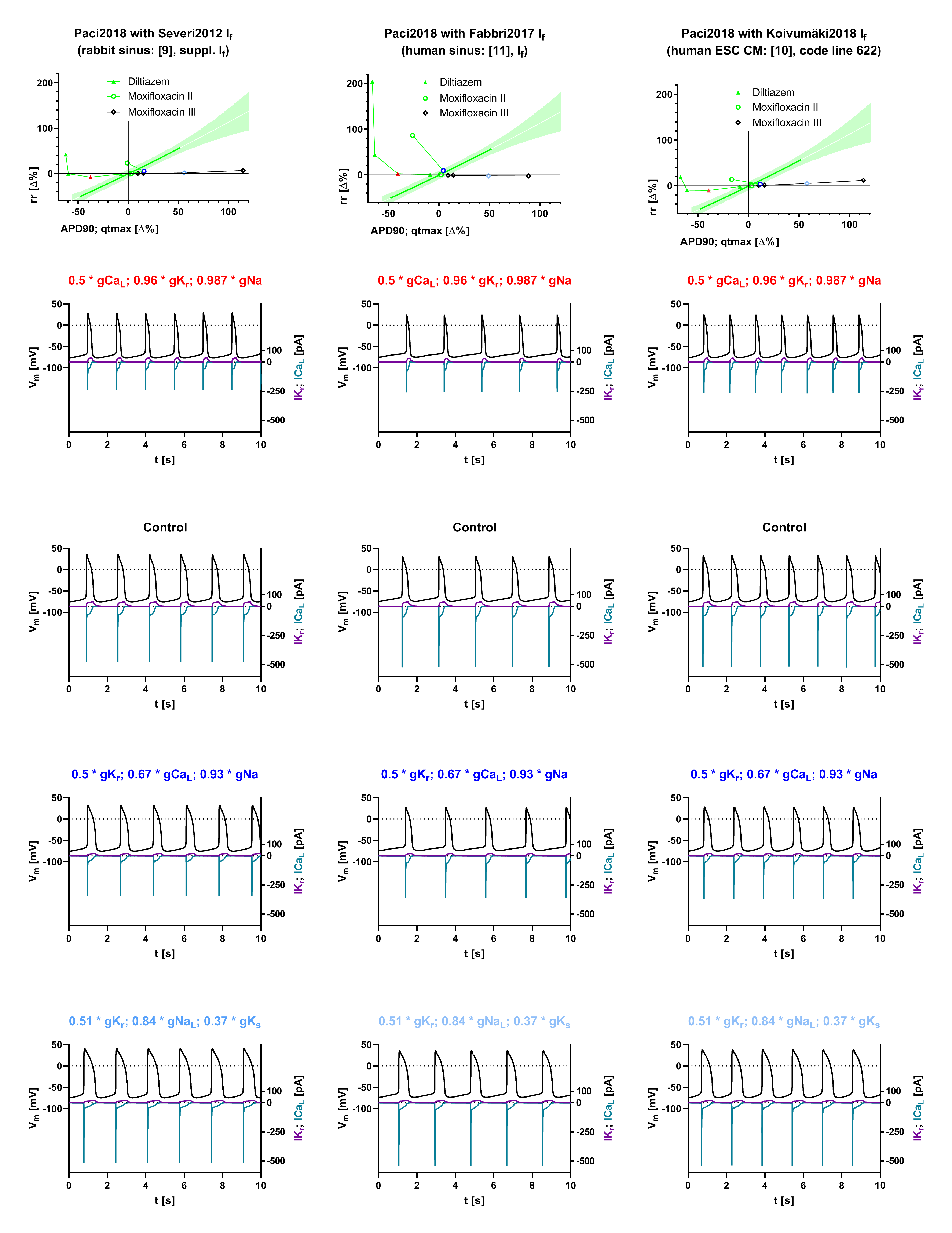

Supplement: S2 Fig — If in the model from [7] was substituted with the equations for If from [9] (Severi2012, left column), [11] (Fabbri2017, middle column), and [11] (Koivumäki2018, right column). First row: Shaded areas: 90% prediction intervals for polynomial fit (solid lines) of experimental data as published in [1]; solid green lines within shaded areas indicate dynamic range of experimental data. The graphical representation of experimental data is modified from [1] and for illustrative purposes only. Symbols: simulated data; filled upright triangles: reduction of ICaL with Diltiazem (plus IKr and INa as off-targets, see Table 1); open circles: reduction of IKr with Moxifloxacin (plus ICaL and INa as off-targets, see Table 2); open diamonds: reduction of IKr with Moxifloxacin (plus IKs and INa (late) as alternative off-targets, see Table 3). Second to fifth row: Simulated action potentials (top, left y-axis) and currents (bottom, right y-axis) with half inhibition of gCaL with Diltiazem (plus off-targets, second row), no modification of conductances (control, third row),half inhibition of gKr with Moxifloxacin (plus off-targets according to Table 2, fourth row), and half inhibition of gKr with Moxifloxacin (plus off-targets according to Table 3, fifth row). Scale factors given in the titles of the panels correspond to the symbols in the first row plots with same color. When two different currents are plotted on a common y-axis, the trace color corresponds to the color of the axis label. The Matlab code for the generation of the simulation data can be found in the supporting material (S1 Code) along with the parameters for the reference curve fits and the actual tabulated result values are provided in S4 Table. (TIF) [file pone.0221763.s007.tif]

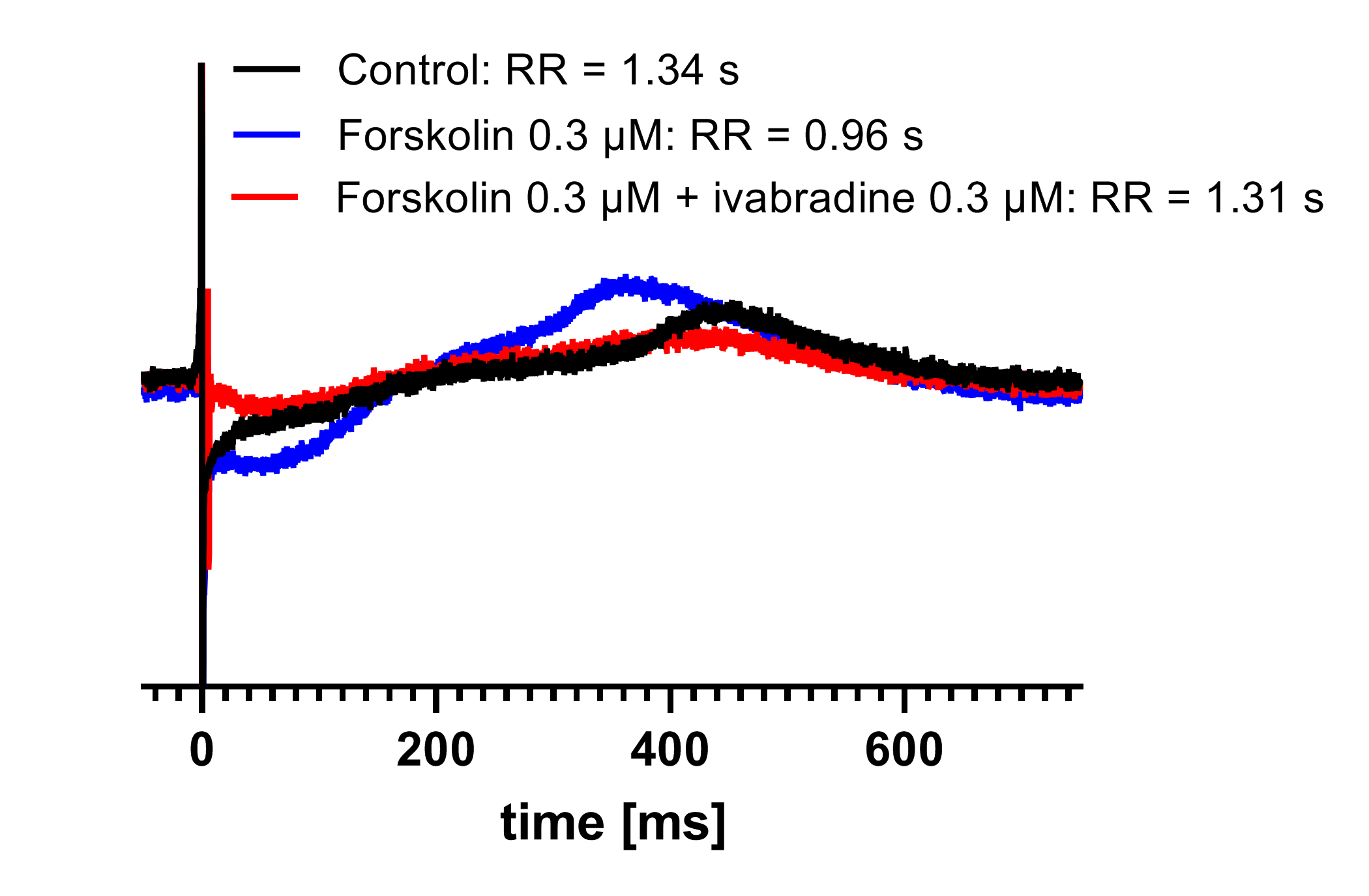

Supplement: S3 Fig — Averaged field potentials recorded from iCells at baseline conditions (Control, black), with 0.3 μM Forskolin (blue) and with 0.3 μM Forskolin + 0.3 μM Ivabradine (red). As expected, Forskolin shortens rr and concomitantly qtmax; after addition of Ivabradine, the reduction of rr is almost completely reversed as is the reduction of qtmax, indicating no relevant residual direct effect on qtmax of Forskolin. (TIF) [file pone.0221763.s008.tif]
